# Guidelines for contributors

**DOI:** 10.2471/BLT.15.990115

**Published:** 2015-01-01

## 1. Scope and editorial policy

### 1.1 Content

The mission of the *Bulletin of the World Health Organization* is “to publish and disseminate scientifically rigorous public health information of international significance that enables policy-makers, researchers and practitioners to be more effective; it aims to improve health, particularly among disadvantaged populations”.

The *Bulletin* welcomes a variety of unsolicited manuscripts (see below, 1.1.1.). These are initially screened in house for originality, relevance to an international public health audience and scientific rigour. If they pass the initial screening, they are sent to peer reviewers whose opinions are taken into account by the journal’s editorial advisers when they decide whether to accept a manuscript for publication. Accepted papers are subject to editorial revision, which may involve substantive changes, shortening or restructuring the text and deleting superfluous tables and figures. The word limits given for each type of contribution do not include the abstract (where applicable), tables, boxes, figures and references or appendices, if any. The principal types of manuscripts are outlined below.

#### 1.1.1. Unsolicited manuscripts

We welcome unsolicited submissions to the Research, Systematic reviews, Policy & practice, Lessons from the field and Perspectives sections of the *Bulletin*. All manuscripts destined for the first four of these sections must include two paragraphs indicating what they add to the literature. The paragraphs should briefly explain:

- what was already known about the topic concerned;
- what new knowledge the manuscript contributes.

##### Research

Research, methodologically rigorous, of relevance to international public health. Formal scientific presentations having not more than 3000 words and 50 references, plus a structured abstract (see below, 2.7); peer reviewed. As clear reporting is needed for readers and reviewers when judging the quality of research, studies should comply with the relevant reporting guidelines, available on the EQUATOR Network web site, at: http://www.equator-network.org/about-equator/equator-publications0/equator-network-publications-2010. Intervention trials as defined by WHO (i.e. “any research study that prospectively assigns human participants or groups of humans to one or more health-related interventions to evaluate the effects on health outcomes”) require registration in a public trials registry acceptable to the International Committee of Medical Journal Editors (ICMJE) before submission, and the registration number must be provided at the end of the abstract. Acceptable registries are listed at: http://www.icmje.org/faq_clinical.html. Web publication constitutes prior publication. This includes institutional web sites that are open to the general public.

##### Systematic reviews 

Exhaustive, critical assessments of published and unpublished studies (grey literature) on research questions concerning interventions, policies or practices in public health, with meta-analysis when feasible. Not more than 3000 words plus a structured abstract (see below, 2.7); the number of references in accordance with the scope of the review; peer reviewed. How studies were included and excluded should be illustrated in a flow diagram. Authors should strictly follow the reporting guidelines for systematic reviews and meta-analyses (PRISMA) available at: http://www.equator-network.org/reporting-guidelines/preferred-reporting-items-for-systematic-reviews-and-meta-analyses-the-prisma-statement. 

##### Policy & practice

Analytical assessments, debates or hypothesis-generating papers; not more than 3000 words and 50 references, plus a non-structured abstract (see below, 2.7); peer reviewed.

##### Lessons from the field

Papers that capture experiences and practice gained in solving specific public health problems in developing countries. Convincing evidence of effect should be provided. Not more than 1500 words and 15 references, plus a structured abstract (see below, 2.7); not more than one table and one figure; must include one box listing three lessons learnt; peer reviewed (see: http://www.who.int/bulletin/volumes/84/1/3.pdf). 

##### Perspectives

Views, hypotheses or discussions (with a clear message) surrounding an issue of public health interest; up to 1500 words, no more than six references; peer reviewed.

#### 1.1.2. Commissioned manuscripts

The categories of articles shown below are normally commissioned by the editors. Authors wishing to submit an unsolicited manuscript for one of these categories should first contact the editorial office (see below, 2.1).

##### Editorials

Authoritative reviews, analyses or views of an important topic related to a theme or to one or more papers published in a given issue; not more than 800 words, maximum 12 references.

##### Commentaries

Explanatory or critical analyses of individual articles; not more than 800 words and 12 references.

##### Round tables

A base paper on a controversial current topic in public health (not more than 2000 words and an abstract) is the core of a debate by several discussants invited to contribute not more than 500 words each.

### 1.2 Ethical issues

The World Health Organization (WHO) publishes the results of research involving human subjects only if fully compliant with ethical principles, including the provisions of the World Medical Association Declaration of Helsinki (as amended by the 59th General Assembly, Seoul, the Republic of Korea, October 2008; available at: http://www.wma.net/en/30publications/10policies/b3/17c.pdf) and with the additional requirements, if any, of the country in which the research was carried out. Any manuscript describing the results of such research must contain a clear statement to this effect and should specify that the free and informed consent of the subjects or their legal guardians was obtained and that the relevant institutional or national ethics review board approved the investigation. The *Bulletin* is a member of the Committee on Publication Ethics (COPE; see: http://publicationethics.org). Issues involving publication ethics may be referred to this committee by the editors. WHO Ethics Review Committee clearance is required for papers that report research supported by WHO or that are authored or co-authored by someone who was a WHO staff member while the research was conducted.

### 1.3 Competing interests

A competing interest arises when a professional judgement concerning a primary interest (such as patients’ welfare or the validity of research) may be influenced by a secondary interest (such as financial gain or personal rivalry). We ask all authors to disclose at the time of submission any competing interests that they may have. Examples of competing interests may be found at: http://www.icmje.org. Further information on competing interests is available at: http://www.who.int/bulletin/volumes/83/9/645.pdf.

### 1.4 Funding

Authors should identify the sources that funded the work undertaken, affirm not having entered into an agreement with the funder that may have limited their ability to complete the research as planned, and indicate that they have had full control of all primary data.

### 1.5 Appeals process

Authors of rejected papers can appeal against the decision by following the procedures outlined in an editorial published in the *Bulletin* (see: http://www.who.int/bulletin/volumes/83/9/645.pdf).

## 2. Preparation and submission of manuscripts

### 2.1 Correspondence

Manuscripts should be submitted to the *Bulletin* via our submissions web site (http://submit.bwho.org), where full instructions are given. Queries about online submissions should be sent to: bulletin.submit.ask@who.int. Authors requiring assistance with online submission can contact the editorial office.

### 2.2 Uniform requirements

Manuscripts should be prepared in accordance with the *ICMJE recommendations for the conduct, reporting, editing and publication of scholarly work in medical journals*. The complete document, updated in August 2013, is available at: http://www.icmje.org/urm_main.html.

### 2.3 Languages

Manuscripts should be submitted in English and will be published in that language in the *Bulletin*; the abstracts are translated into Arabic, Chinese, French, Russian and Spanish.

### 2.4 Authorship

On the manuscript’s title page authors should give their full names and the name, city and country of their institutions. The corresponding author must also provide a full postal address, which will be published with the e-mail address unless otherwise requested. Academic titles and the names of departments and subdepartments are unnecessary and are discouraged for reasons of space. If an author has several affiliations, only the most important one should be provided. The criteria for authorship described in the *ICMJE recommendations* (see above, 2.2) must be rigorously observed. Each author should have participated sufficiently in the work being reported to take public responsibility for the paper’s content and should describe in detail on the online submission system (not within the manuscript itself) his or her particular contribution. The *Bulletin* encourages submissions from authors in developing countries, and in line with this policy at least one author should have a professional affiliation in the country where the study was conducted.

### 2.5 Licence for publication

If a manuscript is accepted for publication, the author(s) will be asked to sign a statement granting exclusive licence for publication (not copyright) to the WHO. A copy of the statement is available at: http://submit.bwho.org/journals/bullwho/forms/licence.pdf. Authors are responsible for obtaining permission to reproduce in their articles any material enjoying copyright protection. They should send the letter granting such permission to the editorial office when they submit their papers.

### 2.6 Figures, tables and boxes

These should be used only to enhance the understanding of the text, not to repeat what can be clearly communicated within the text. All tables, figures and boxes should be numbered consecutively (e.g. Fig. 1, Table 1 and Box 1).

### 2.7 Abstracts

Abstracts should highlight the text’s most important points and should be provided for the following types of papers: Research, Systematic reviews, Policy & practice, base papers for Round tables and Lessons from the field. The abstract should not exceed 250 words. It appears in English at the beginning of the paper and in Arabic, Chinese, French, Russian and Spanish between the end of the text and the reference list. Structured abstracts are required for Research papers and Systematic reviews (Objective, Methods, Findings, Conclusion) and for Lessons from the field papers (Problem, Approach, Local setting, Relevant changes, Lessons learnt). 

### 2.8 Bibliographic references

Reference citations should be numbered consecutively as they occur in the text and references should be listed in accordance with the *ICMJE recommendations* (http://www.icmje.org/manuscript_a.html). The accuracy of all references is the authors’ responsibility and authors are also responsible for dating access to URLs, providing a record of when they were active.

### 2.9 Maps

Papers should contain no maps unless an important finding cannot be conveyed without them or unless they are needed to make an essential point. Maps that show international borders, partially or in full, must be created from one of the following sources, approved by the United Nations: http://www.un.org/Depts/Cartographic/english/htmain.htm, http://www.unsalb.org or http://apps.who.int/tools/geoserver and the vectorial EPS (Encapsulated PostScript) file must be submitted.

